# Synthesis and Structure-Activity Relationships of Novel Amino/Nitro Substituted 3-Arylcoumarins as Antibacterial Agents

**DOI:** 10.3390/molecules18021394

**Published:** 2013-01-24

**Authors:** Maria J. Matos, Saleta Vazquez-Rodriguez, Lourdes Santana, Eugenio Uriarte, Cristina Fuentes-Edfuf, Ysabel Santos, Angeles Muñoz-Crego

**Affiliations:** 1 Departamento de Química Orgánica, Facultad de Farmacia, Universidad de Santiago de Compostela, 15782 Santiago de Compostela, Spain; E-Mails: svre77@gmail.com (S.V.-R.); lourdes.santana@usc.es (L.S.); eugenio.uriarte@usc.es (E.U.); 2 Departamento de Microbiología y Parasitología, Facultad de Biología, Universidad de Santiago de Compostela, 15782 Santiago de Compostela, Spain; E-Mails: cristina.fuentesedfuf@gmail.com (C.F.-E.); ysabel.santos@usc.es (Y.S.); a.munoz.crego@usc.es (A.M.-C.)

**Keywords:** amino/nitro substituted 3-arylcoumarins, Perkin reaction, antibacterial assays, *S. aureus*, *E. coli*

## Abstract

A new series of amino/nitro-substituted 3-arylcoumarins were synthesized and their antibacterial activity against clinical isolates of *Staphylococcus aureus* (Gram-positive) and *Escherichia coli* (Gram-negative) was evaluated. Some of these molecules exhibited antibacterial activity against *S. aureus* comparable to the standards used (oxolinic acid and ampicillin). The preliminary structure-activity relationship (SAR) study showed that the antibacterial activity against *S. aureus* depends on the position of the 3-arylcoumarin substitution pattern. With the aim of finding the structural features for the antibacterial activity and selectivity, in the present manuscript different positions of nitro, methyl, methoxy, amino and bromo substituents on the 3-arylcoumarin scaffold were reported.

## 1. Introduction

**Figure 1:**
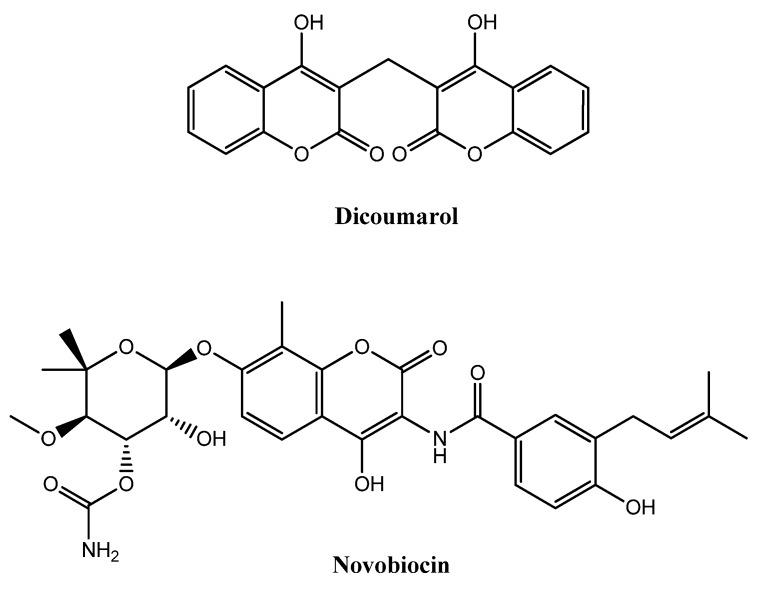
Chemical structures of dicoumarol and novobiocin.

Drug discovery is considered a complex and slow activity. However, with the intention of discovering new chemical entities in a new and more efficient way, medicinal chemistry researchers have developed new approaches and methods. Some of the new products are based on diversity molecules found and extracted from natural sources. Coumarins are a large family of compounds of natural or synthetic origin, associated with remarkable pharmacological activities [1,2]. They occur naturally in plants and microorganisms and approximately 1,000 coumarin derivatives have been isolated from over 800 species [2]. The fused heterocyclic framework of coumarins has been used as a prototype scaffold for the synthesis of a wide variety of analogues in order to study and improve their biological properties. Its structural variety is responsible for the important place they occupy in the realm of natural products and synthetic organic chemistry [2,3]. Some coumarins have been studied for their antimicrobial [4,5,6,7], cardioprotective [8], anticancer [9,10], antioxidative [11] and enzymatic inhibition properties [12,13,14,15,16,17]. Goth described the antibacterial properties of coumarins for the first time in 1945 when he studied the dicoumarol (Figure 1) [18]. Some other coumarin derivatives have proven to display antibacterial and antifungal activities [19,20,21,22]. A study of coumarin derivatives substituted on the pyrone ring indicated that 3-carboxyl derivatives show significant activities [23]. Different 4-substituted coumarins, such as 4-chlorocoumarins, exhibit also an interesting antimicrobial profile [24,25]. Novobiocin (Figure 1), chlorobiocin and coumermycin A_1_ are important natural occurring antibiotics in which the coumarin nucleus is present in their skeleton [26].

The most active of them is novobiocin, isolated from *Streptomyces* *niveus* and which is mainly active against Gram-positive bacteria. Although they antagonize the B subunit of the essential *E. coli* DNA gyrase supertwisting activity *in vitro* and the bacterial multiplication, they are not used in the clinic due to their relatively weak activity against Gram-negative bacteria, side effects and poor water solubility [4,26]. On the other hand, these three examples are antibiotics, which present activity against methicillin-resistant *S. aureus* (MRSA) [26]. The number of multidrug-resistant (MDR) bacteria is increasing and the Gram-positive MRSA are of particular importance. The ability of *S. aureus *species to develop resistance to virtually all antibiotics is a major concern and the discovery of new antimicrobial agents is essential to combat this problem [27]. MRSA are often an important problem for severe infections in patients, especially if they are immunosuppressed, during long stays in hospitals [27]. The dramatic worldwide increase of dangerous infections by resistant and multi-resistant microbes makes, therefore, the search of new molecules and new chemical entities an important topic in medicinal chemistry [28,29]. As the ideal drug candidate has not been attained, an intensive search for new and innovative antimicrobials is still needed. Unfortunately, due to economic reasons, pharmaceutical companies have considerably decreased this effort in recent years [30].

## 2. Results and Discussion

**Scheme 1:**
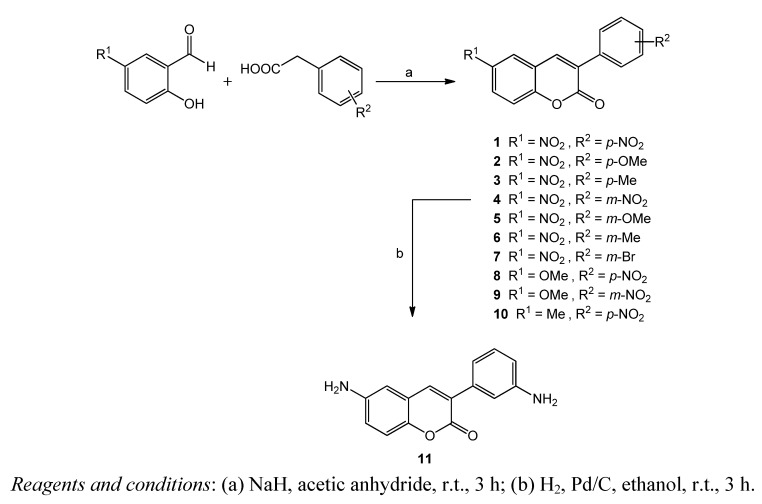
Synthesis of 3-arylcoumarins (**1**-**11**).

Based on previous findings in the area [24,25], and in our experience with substituted 3-arylcoumarins [15,31,32], in the present work we proposed the synthesis and antibacterial evaluation of a series of different substituted amino/nitro 3-arylcoumarins (Scheme 1). With the aim of finding the structural features for the antibacterial activity and selectivity, we decided to explore the importance of the nature and position of small groups (methoxy, bromo, nitro, amino and methyl substituents) into both coumarin nucleus and 3-aryl ring (Scheme 1).

New derivatives **2**, **4**–**7** and **9**–**11**, as well as the already described compounds **1**, **3** and **8** [33,34,35,36], were efficiently synthesized according to the synthetic protocol outlined in Scheme 1. 3-Arylcoumarins **1**–**10** were synthesized in a dry Schlenk tube, in presence of sodium hydride and with acetic anhydride as solvent, at room temperature for three hours. The reaction mixture was purified by flash chromatography, using hexane/ethyl acetate as eluent in a proportion of 9:1. Starting from different commercially available substituted salicylaldehydes and the respectively substituted phenylacetic acids, we obtained ten derivates in good yields (60–75%). The derivative **11** was obtained from **4**, in ethanol, with palladium/carbon as catalyst and under hydrogen atmosphere for three hours. The reaction mixture was purified by flash chromatography, using hexane/ethyl acetate as eluent, in a proportion of 9:1. We obtained the desired derivate in good yield (80%). Then, two different methodologies were employed for the antibacterial evaluation of these compounds: disk diffusion test and microdilution method. Clinical antimicrobial drugs–oxolinic acid and ampicillin–were used as positive controls. The inhibition zones for growth inhibitory effects of the new compounds and controls were measured in millimetres (Table 1, method A). The minimum inhibitory concentrations (MICs) of the new compounds and controls were measured in microgram/millilitre (Table 1, method B).

**Table 1:** *In vitro* antibacterial activity ^a^ of amino/nitro substituted 3-arylcoumarins **1**–**11** (**A**) expressed as inhibition zones (mm) ^b^ and (**B**) expressed as MICs (µg/mL).

Compounds	Method A (mm)		Method B (µg/mL)	
	*S. aureus*	*E. coli*	*S. aureus*	*E. coli*
1	19	NA	128	-
2	25	NA	64	-
3	22	NA	32	-
4	22	NA	32	-
5	25	NA	64	-
6	32	NA	8	-
7	14	NA	256	-
8	NA	NA	>512	-
9	NA	NA	>512	-
10	NA	NA	>512	-
11	18	NA	128	-
Oxolinic acid	26	31	2	<1
Ampicillin	32	23	2	8

^a^ These results are average results of three experiments; ^b^ The compounds were used at the concentration of 100 µg/disk and the inhibition zones are stated in mm; NA = not active; diameter of inhibition zone ≤ 5 mm.

The obtained inhibition zones revealed that all the 6-nitro derivatives (compounds **1**–**7**) presented antibacterial activity against *S. aureus*, showing inhibition zones ranging between 14 and 32 mm. The amino derivative **11** presented activity against the same bacterial strain. The 3-arylcoumarins nitro substituted only in the 3-aryl ring (compounds **8**–**10**) were inactive against *S. aureus *in the *in vitro* disk diffusion test assays, independently of the relative position of this nitro group (*para* or *meta *positions). From the data, it is shown that compound **6** and ampicillin presented the same inhibition zone against *S. aureus* (32 mm) and a higher inhibition zone than the oxolinic acid (26 mm). Hence, the preliminary experimental data revealed that some of the tested compounds showed a very interesting activity profile against *S. aureus* when compared with the standards ampicillin and oxolinic acid. In addition, the different nature and position of the substituent in the coumarin scaffold seems to influence the antibacterial activity. 

In order to deeply study the antibacterial activities and quantify the previously mentioned information, MICs of compounds **1**–**11** against *S. aureus* were also determined. This second methodology (method B) is used to clarify and refine the previously results obtained by method A, which could be influenced by differences in the diffusion of the compounds. This study was performed against the same bacteria strain, by a microdilution assay with serial dilutions (from 512 to 1 μg/mL) of the synthesized and the reference compounds. MICs of compounds **1**–**11** against *E. coli* were not evaluated, taking into account the lack of inhibition zones in the previous disk diffusion test (inhibition zones ≤ 5 mm). Analysing the MICs results for *S. aureus*, it is clear that the presence of a nitro substituent at the six position of coumarin moiety (compounds **1**–**7**) is more beneficial than its presence only at *meta* or *para* positions of the 3-aryl ring (compounds **8**–**10**). 

Compounds with nitro substituents either in the coumarin moiety and in the aryl group (compounds **1** and **4**) are not better than compounds where the nitro in the aryl ring is substituted for a methyl or a methoxy group (compounds **2** and **3** compared to compound **1**, and compounds **5** and **6** compared to compound **4**). However, compound **4** (MIC = 32 µg/mL), with the nitro substituent in *meta* position of the 3-aryl ring, is better than compound **1** (MIC = 128 µg/mL), with the same substituent in *para* position. The presence of a methyl group in the *meta* position is the best substitution when one nitro substituent is present at six position of the coumarin moiety (compound **6**, MIC = 8 µg/mL). In general, *meta* substitutions are more favourable than *para*, being the *meta*-methyl substituent the most active, followed by *meta*-nitro and finally *meta*-methoxy. There is one exception to this conclusion. The introduction of a bromo atom at the same position was the worst substitution of the studied 3-aryl-6-nitrocoumarins (compound **7**, MIC = 256 µg/mL). A *para*-methyl substituent in the 3-aryl ring (compound **3**, MIC = 32 µg/mL) is the best substitution in this *para* position. Therefore, the *para*-methyl substitution is better than the *para*-nitro and *para*-methoxy substitutions. Comparing the two positions in the 3-aryl ring, the substitutions by methyl groups are more favourable for the desired activity. The substitution of nitro by amino groups significantly decreases the activity of the described compounds (comparing compounds **4** and **11**).

In order to evaluate if there was a correlation between the activity and the lipophilicity, and to better understand the overall properties of the described compounds, logP (octanol/water partition coefficient) values were calculated using the Molinspiration property calculation program [37]. The results are presented in Table 2.

**Table 2:** LogP (octanol/water partition coefficient) values calculated for the amino/nitro substituted 3-arylcoumarins **1**–**11**^a^.

Compounds	LogP
1	3,631
2	3,729
3	4,120
4	3,607
5	3,705
6	4,096
7	4,457
8	3,729
9	3,705
10	4,120
11	1,841

^a^ The data was determined with Molinspiration calculation software [37].

Analysing the obtained data, it can be concluded that there are no linear correlations between the lipophilicity and the activity of the compounds. Compounds **5** and **6** are two of the best compounds against *S. aureus*, presenting intermediate, but different, logP values (3.705 and 4.096 respectively). The compounds with the lowest (compound **11**, 1.841) and the highest (compound **7**, 4.457) logP presented activity. Compound **9**, with the same logP as compound **5**, resulted to be inactive. Also, compound **10**, with the same lipophilicity profile as compound **3** (logP = 4.12), resulted to be inactive. Therefore, there are no clear correlation between activity and logP values. It is important to highlight that the calculated logP values for all the described compounds are lower than 5.

## 3. Experimental

### 3.1. Chemistry

Merck supplied the entire chemicals used in the synthesis. Melting points (mp) are uncorrected were determined using a Reichert Kofler thermopan or in capillary tubes on a Büchi 510 apparatus and are uncorrected. ^1^H-NMR (300 MHz) and ^13^C-NMR (75.4 MHz) spectra were recorded with a Bruker AMX spectrometer. Chemical shifts (δ) are expressed in parts per million (ppm) using TMS as an internal standard. Spin multiplicities are given as s (singlet), d (doublet), dd (doublet of doublets) and m (multiplet). Mass spectrometry was carried out with a Kratos MS-50 or a Varian MAT-711 spectrometer. Elemental analyses were performed by a Perkin-Elmer 240B microanalyzer and were within ±0.4% of calculated values in all cases. Flash Chromatography (FC) was performed on silica gel (Merck 60, 230–400 mesh); analytical TLC was performed on precoated silica gel plates (Merck 60 F254).

General Procedure for the preparation of 3-arylcoumarins **1**–**10**. In a 20 mL dry Schlenk tube, to a solution of the conveniently substituted salicylaldehyde (2.46 mmol) and the arylacetic acid (2.46 mmol), in acetic anhydride (6 mL), NaH (2.46 mmol) was added in small portions, and the reaction mixture was stirred for 3 hours, at room temperature. The obtained crude was filtered and washed with diethyl ether. The solid was then purified by flash chromatography (hexane/ethyl acetate 9:1) to give the desired coumarins **1**–**10**. 

*3-(4′-Methoxyphenyl)-6-nitrocoumarin* (**2**). Pale yellow solid, 75% yield. Mp: 62–63 °C. ^1^H-NMR (CDCl_3_) δ (ppm): 3.80 (s, 3H, OCH_3_), 7.05 (d, 2H, H-3′, H-5′, *J* = 9.45 Hz), 7.62–7.69 (m, 3H, H-2′, H-6′, H-8), 8.36 (s, 1H, H-4), 8.73 (d, 2H, H-5, H-7, *J* = 2.93 Hz). ^13^C-NMR (CDCl_3_) δ (ppm): 55.9, 116.4, 119.7, 127.9, 128.4, 128.6, 129.2, 132.2, 132.5, 134.3, 139.1, 139.4, 151.5, 155.8, 161.2. ESI-MS *m/z*: 297 (M^+^, 100). Anal. Calcd. for C_16_H_11_NO_5_: C, 64.65; H, 3.73; Found: C, 64.67; H, 3.76.

*3-(3′-Nitrophenyl)-6-nitrocoumarin* (**4**). Pale yellow solid, 69% yield. Mp: 87–88 °C. ^1^H-NMR (CDCl_3_) δ (ppm): 7.57–7.63 (m, 2H, H-8, H-6′), 7.71–7.77 (m, 1H, H-5′), 8.15 (s, 1H, H-4), 8.42-8.63 (m, 4H, H-4′, H-2, H-5, H-7). ^13^C-NMR (CDCl_3_) δ (ppm): 118.1, 120.2, 123.1, 123.3, 124.3. 124.5, 124.7, 129.4, 133.6, 135.1, 141.2, 144.6, 147.9, 159.2, 160.5. ESI-MS *m/z*: 312 (M^+^, 100). Anal. Calcd. for C_15_H_8_N_2_O_6_: C, 50.70; H, 2.58. Found: C, 50.72; H, 2.60.

*3-(3′-Methoxyphenyl)-6-nitrocoumarin* (**5**). Pale yellow solid, 71% yield. Mp: 53–54 °C. ^1^H-NMR (CDCl_3_) δ (ppm): 3.71 (s, 3H, OCH_3_), 6.80 (s, 1H, H-2′), 7.17–7.22 (m, 3H, H-4′, H-5′, H-6′), 7.57 (d, 1H, H-8, *J* = 6.5 Hz), 7.77 (s, 1H, H-5), 8.19 (d, 1H, H-7, *J* = 6.5 Hz), 8.39 (s, 1H, H-4). ^13^C-NMR (CDCl_3_) δ (ppm): 55.4, 84.5, 112.4, 115.5, 122.0, 123.5, 125.8, 126.8, 129.5, 129.7, 136.9, 145.7, 153.2, 159.6, 168.7, 173.0. ESI-MS *m/z*: 297 (M^+^, 100). Anal. Calcd. for C_16_H_11_NO_5_: C, 64.65; H, 3.73. Found: C, 64.63; H, 3.70.

*3-(3′-Methylphenyl)-6-nitrocoumarin* (**6**). Pale yellow solid, 69% yield. Mp: 55–56 °C. ^1^H-NMR (CDCl_3_) δ (ppm): 2.25 (s, 3H, CH_3_), 6.90–7.30 (m, 5H, H-2′, H-4′, H-5′, H-6′, H-8), 7.75 (s, 1H, H-4), 8.44 (d, 1H, H-7, *J* = 2.8 Hz), 8.62 (d, 1H, H-5, *J* = 2.8 Hz). ^13^C-NMR (CDCl_3_) δ (ppm): 21.5, 118.4, 123.3, 124.5, 124.6, 124.8, 125.9, 126.6, 128.3, 128.5, 132.7, 138.4, 141.1, 144.6, 159.3, 160.9. ESI-MS *m/z*: 281 (M^+^, 100). Anal. Calcd. for C_16_H_11_NO_4_: C, 68.32; H, 3.94. Found: C, 68.31; H, 3.92.

*3-(3′-Bromophenyl)-6-nitrocoumarin* (**7**). Pale yellow solid, 60% yield. Mp: 240–241 °C. ^1^H-NMR (CDCl_3_) δ (ppm): 7.12–7.26 (m, 2H, H-4′, H-5′), 7.46–7.71 (m, 5H, H-2′, H-6′, H-5, H-7, H-8), 7.78 (s, 1H, H-4). ^13^C-NMR (CDCl_3_) δ (ppm): 117.9, 122.7, 123.1, 124.5, 124.6, 124.8, 127.9, 129.5, 129.7, 130.8, 134.6, 140.7, 144.6, 159.1, 161.1. ESI-MS *m/z*: 345 (M^+^, 100). Anal. Calcd. for C_15_H_8_BrNO_4_: C, 52.05; H, 2.33. Found: C, 52.01; H, 2.30. 

*6-Methoxy-3-(3′-nitrophenyl)coumarin* (**9**). Pale yellow solid, 73% yield. Mp: 84–85 °C. ^1^H-NMR (CDCl_3_) δ (ppm): 3.77 (s, 3H, OCH_3_), 7.01–7.10 (m, 2H, H-5, H-7), 7.54–7.59 (m, 2H, H-5′, H-6′), 7.71 (d, 1H, H-8, *J* = 7.6 Hz), 8.06–8.09 (m, 2H, H-2′, H-4′), 8.15 (s, 1H, H-4). ^13^C-NMR (CDCl_3_) δ (ppm): 56.7, 115.0, 116.8, 122.3, 124.9, 128.7, 130.2, 134.2, 135.5, 137.2, 138.0, 143.3, 148.3, 156.8, 167.7, 172.7. ESI-MS *m/z*: 297 (M^+^, 100). Anal. Calcd. for C_16_H_11_NO_5_: C, 64.65; H, 3.73. Found: C, 64.63; H, 3.70.

*6-Methyl-3-(4′-nitrophenyl)coumarin* (**10**). Pale yellow solid, 75% yield. Mp: 100–101 °C. ^1^H-NMR (CDCl_3_) δ (ppm): 2.36 (s, 3H, CH_3_), 7.21 (d, 2H, H-7, H-8, *J* = 8.2 Hz), 7.38 (s, 1H, H-5), 7.63–7.70 (m, 2H, H-2′, H-6′), 7.99–8.20 (m, 2H, H-3′, H-5′), 8.38 (s, 1H, H-4). ^13^C-NMR (CDCl_3_) δ (ppm): 20.6, 122.6, 123.9, 125.1, 127.9, 130.2, 131.4, 136.4, 136.6, 136.8, 137.2, 148.1, 149.3, 170.2, 190.6. ESI-MS *m/z*: 281 (M^+^, 98). Anal. Calcd. for C_16_H_11_NO_4_: C, 68.32; H, 3.94. Found: C, 68.38; H, 3.99. 

#### Procedure for the Preparation of 3-(3′-Aminophenyl)-6-Aminocoumarin (***11***)

The previously prepared 3-(3′-nitrophenyl)-6-nitrocoumarin (**4**, 2.46 mmol) was dissolved in ethanol (5 mL) and a catalytic amount of Pd/C was added to the mixture. The solution was stirred, at room temperature, under a H_2_ atmosphere, for 3 h. After completion of the reaction, the mixture was filtered to eliminate the catalyst. The obtained crude was then purified by flash chromatography (hexane/ethyl acetate 9:1) to give the desired coumarin **11** as a white solid in 80% yield. Mp: 105–106 °C. ^1^H-NMR (CDCl_3_) δ (ppm): 4.02 (s, 2H, NH_2_), 6.50–6.55 (m, 1H, H-4′) 6.56 (s, 1H, H-2′), 6.69–6.73 (m, 1H, H-6′) 6.80 (dd, 2H, H-5, H-7, *J* = 7.8, *J* = 2.4 Hz), 7.30–7.38 (m, 2H, H-5′, H-8), 8.4 (s, 1H, H-4). ^13^C-NMR (CDCl_3_) δ (ppm): 114.9, 115.7, 116.7, 120.9, 123.6, 125.4, 127.2, 128.6, 133.7, 135.8, 136.2, 143.3, 145.5, 149.7, 161.9. ESI-MS *m/z*: 252 (M^+^, 100). Anal. Calcd. for C_15_H_12_N_2_O_2_: C, 71.42; H, 4.79. Found: C, 71.45; H, 4.82.

*Disk Diffusion Test:* The antimicrobial activity of the compounds was assayed by the disk diffusion method, following the procedures of the Clinical and Laboratory Standards Institute (CLSI, 2006). The inoculum was prepared as a saline suspension of colonies from a 24 h Mueller Hinton Agar (MHA) (Cultimed, Barcelona, Spain) plate culture of the microorganisms, containing approximately 1 × 10^8^ colony forming units (CFU)/mL (OD_620_ of 0,09). Colony counts on inoculum suspension were verified by the plate dilution method using MHA plates and counting the bacterial colonies produced. The entire surface of the MHA plate was inoculated by streaking with the swab containing the inoculum. Antimicrobial solution (concentration of 10000 µg/mL) was prepared using DMSO as solvent. Sterile disks of 6 mm diameter (Liofilchem, Roseto degli Abruzzi, Italy) embedded in the drug at a final concentration of 100 µg/disk were kept on agar surface. Sterile disks embedded with DMSO were used as a negative control. The plates were incubated at 37 °C for 24 h. Zones of inhibition were measured in millimeter. 

*Disk Minimum Inhibitory Concentrations* (*MIC*s): The MICs were evaluated using the broth microdilution method, following the procedures of the Clinical and Laboratory Standards Institute (CLSI, 2006). Broth microdilution tests were performed with 96 sterile flat-bottom microtiter plates (Becton Dickinson Labware Europe, Madrid, Spain). Antimicrobials were serially diluted (512 to 1 μg/mL) in Mueller-Hinton Broth (MHB) (Cultimed) and then one hundred microliters of each dilution were deposited into each well. The inoculum was prepared by making a MHB suspension of colonies from a 24 h MHA (Cultimed) plate culture of the microorganisms, containing approximately 1 × 10^8^ colony forming units (CFU)/mL (OD_620_ of 0,09). The inoculum was diluted 1:100 in MHB to yield 1 × 10^6 ^CFU/mL. Ten microliters of this suspension was inoculated into the each well. Colony counts on inoculum suspension were verified by the plate dilution method using MHA plates and counting the bacterial colonies produced. MHB with and without inoculum was used as growth-control and negative control, respectively. The plates were incubated at 37 °C for 24 h. The MIC was established comparing the amount of growth in the wells containing the antimicrobial agents with the amount of growth in the growth-control wells both with the unaided eye and by using a photometric device (OD_620_).

## 4. Conclusions

In conclusion, in the present study it was shown that eight out of the eleven synthesized amino/nitro-substituted 3-arylcoumarins have inhibitory activity against *S. aureus*. MIC determinations proved that the tested compounds presented different profiles against *S. aureus* due to their substituents, being 3-(3′-methylphenyl)-6-nitrocoumarin (**6**) the best one. A nitro substituent at the 6-position of the coumarin moiety seems to be essential for the antibacterial activity of this kind of compounds. The introduction of an amino substituent seems to decrease the described activity. The pharmacological potential of these amino/nitro-substituted 3-arylcoumarins confirms that this scaffold can be effectively optimized into a candidate for the treatment of some bacterial infectious diseases.
